# Identification of surface defects and *in situ* lattice reconstruction of upconversion nanoparticles

**DOI:** 10.1039/d6sc03950b

**Published:** 2026-07-27

**Authors:** Fenglin Wang, Xiaoyong Huang, Yunfei Shang, Jun Zeng, Xiangyu Pan, Fei Han, Yongtao Liu, Shuwei Hao, Chunhui Yang, Jiajia Zhou

**Affiliations:** a MIIT Key Laboratory of Critical Materials Technology for New Energy Conversion and Storage, School of Chemistry and Chemical Engineering, Harbin Institute of Technology Harbin 150001 China shangyunfei@hit.edu.cn; b Zhengzhou Research Institute of Harbin Institute of Technology Zhengzhou 450001 China; c School of Electronic and Optical Engineering, Nanjing University of Science and Technology Nanjing 210094 Jiangsu China; d Institute for Biomedical Materials & Devices (IBMD), Faculty of Science, University of Technology Sydney Sydney NSW 2007 Australia Jiajia.Zhou@uts.edu.au

## Abstract

Lanthanide doped upconversion nanoparticles have attracted widespread attention due to their unique and efficient anti-Stokes emission. However, the large specific surface area and high surface quenching rates pose significant challenges in achieving small upconversion nanoparticles with strong emission intensity. Herein, we identify the surface defects that disrupt the crystal lattice periodicity as lanthanide cation vacancies and propose an effective localized lattice reconstruction strategy to block undesired energy transfer from excited states to surface quenching sites in LiYF_4_:Yb,Tm upconversion nanosystems. The improvement in upconversion performance is verified at the single nanoparticle level, eliminating the macroscopic statistical averaging inherent in ensemble measurements using solution- or powder-based systems. Notably, the emission intensity enhancement becomes more pronounced as nanoparticle size decreases. An ∼60-fold emission enhancement of the ^1^G_4_ → ^3^H_6_ transition is achieved on 13.5 nm nanoparticles without increasing the particle size, which demonstrates the significance of suppressing surface quenching for small nanoparticles. This lanthanide ion-assisted post-annealing strategy for surface lattice reconstruction could promote the development of small but bright upconversion nanoparticles for advanced applications.

## Abstract 2

An efficient localized lattice reconstruction strategy is proposed to suppress undesired energy transfer from excited states to surface quenching sites, and the enhancement is verified at single particle level.

## Graphical abstract

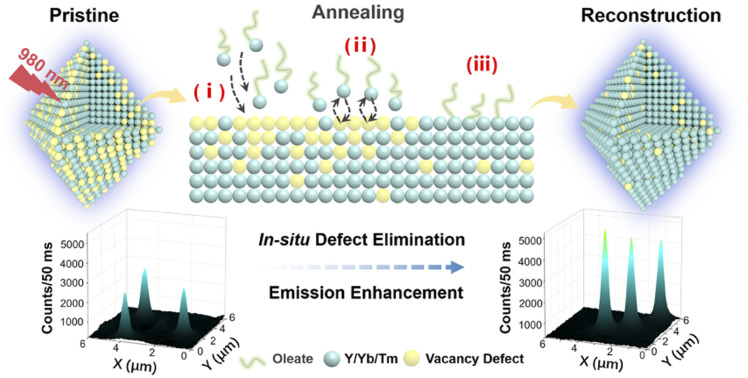

## Introduction

Lanthanide-doped upconversion nanoparticles (UCNPs), due to their unique 4f transitions and efficient anti-Stokes shift luminescence, exhibit tunable emission with long lifetimes, narrow bandwidths, and high photobleaching resistance,^1–3^ demonstrating significant applications in bioimaging,^4–6^ sensing,^7–9^ lasers,^10,11^ anticounterfeiting^12,13^ and information storage.^14–17^ Since the surface lattice defect proportion of small UCNPs is pretty high, which severely quenches the upconversion emission,^18^ researchers have to use large UCNPs to achieve enough brightness for potential applications. The trade-off between size and emission brightness is still a significant challenge that limits the development of UCNPs. Although there are several strategies to enhance the emission intensity, such as inert shell coating,^5,18^ organic molecular surface passivation,^19,20^ dye sensitization,^21–23^ anhydrous synthesis^24–26^ and surface plasmon coupling,^10,27,28^ each of these strategies faces specific limitations. Normally, the core–shell structure typically requires a 6–8 nm inert shell layer to fully suppress detrimental energy transfer, resulting in a substantial increase in size, which limits the potential biological applications.^29,30^ Although organic molecular surface passivation can efficiently enhance the emission intensity (for instance, pyridine-2-carboxylic acid with bidentate coordination capability can form a stable five-membered chelate ring with surface lanthanide ions, thus increasing the luminescence intensity of nanocrystals by up to three orders of magnitude), its poor environmental stability and insufficient biocompatibility make it problematic for use in biological systems.^19,20,31,32^ Anhydrous synthesis demands rigorous water- and oxygen-free reaction environments with extremely harsh experimental operation requirements.^24–26^ Moreover, dye sensitization, photonic crystal engineering, surface plasmon resonance and microlens systems are pretty complex and only applicable to some scenarios.^10,21,23,27,28^ Crucially, the lattice defect-induced quenching sites on the surface have not been fundamentally eliminated.

It is noteworthy that wet chemical annealing offers a promising strategy to eliminate surface defects. Bian *et al.* employed a thermal annealing method to treat KLu_2_F_7_ nanosheets, revealing the restoration of surface related defects and achieving an approximately 10-fold enhancement in photoluminescence intensity.^33^ However, the generalizability and reliability may be limited by the inherent anisotropic morphology and non-uniform size distribution of the as-prepared nanosheets. Then, Chen *et al.* demonstrated that the elimination of point defects could significantly enhance emission intensity through treating the NaGdF_4_@NaYF_4_ core–shell nanoparticles with a Y^3+^-assisted annealing strategy.^34^ However, it is difficult to fully distinguish the respective contributions of defect elimination and structural modification to the luminescence enhancement, as the core–shell structure also introduces potential interface lattice mismatch. The direct causal relationship between surface lattice defects and luminescence performance thus remains to be further clarified. Meanwhile, current studies mainly rely on spectral measurements of macroscopic nanoparticle solution or powder, lacking the *in situ* optical characterization at the single nanoparticle level. This gap obscures the precise relationship between specific surface lattice defects and optical performances.

**Fig. 1:**
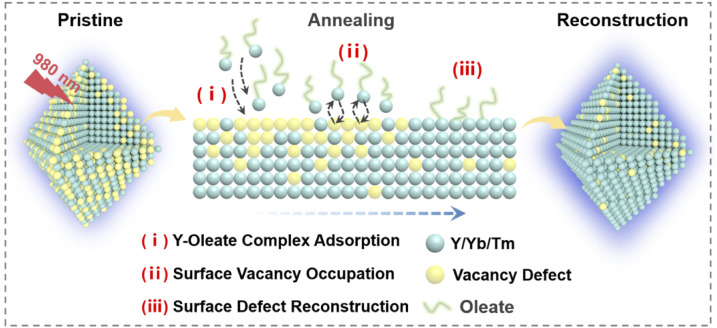
Schematic diagram of Y^3+^ ion assisted surface vacancy defect restoration. (i) Y^3+^-oleate complexes adsorbed onto the nanocrystal surface at high temperature. (ii) Y^3+^ occupies surface cation vacancies and induces lattice rearrangement. (iii) Elimination of surface defects, recovery of lattice structure, and improvement of optical performance.

Herein, we elucidated the type of surface lattice defect and proposed a localized lattice reconstruction strategy to block the detrimental transfer of excited-state energy to surface quenching sites. As shown in Fig. 1, lanthanide ions can interact with the surface vacancies and reconstruct the lattice structure, thereby improving the crystallinity and suppressing surface quenching. To validate this strategy, we treated LiYF_4_:Yb,Tm nanoparticles *via* an ion-assisted wet-chemical annealing strategy and employed a single-particle imaging system combined with aberration-corrected high-angle annular dark-field scanning transmission electron microscopy (AC-HAADF-STEM) to achieve correlated characterization of surface lattice defects and optical performances at the single nanoparticle level. The correlation measurement strategy effectively avoids inherent statistical errors in multi-particle systems, such as solutions or powders, and clarifies the relationship between surface lattice defects and upconversion emission intensity. Meanwhile, as the particle size decreases, the luminescence enhancement becomes more pronounced (from 6 to 60-fold, ^1^G_4_ → ^3^H_6_), further confirming the importance of suppressing surface quenching for boosting the emission of smaller nanoparticles. The proposed surface lattice reconstruction strategy is expected to promote the development of highly efficient UCNPs without increasing the size.

## Results and discussion

**Fig. 2:**
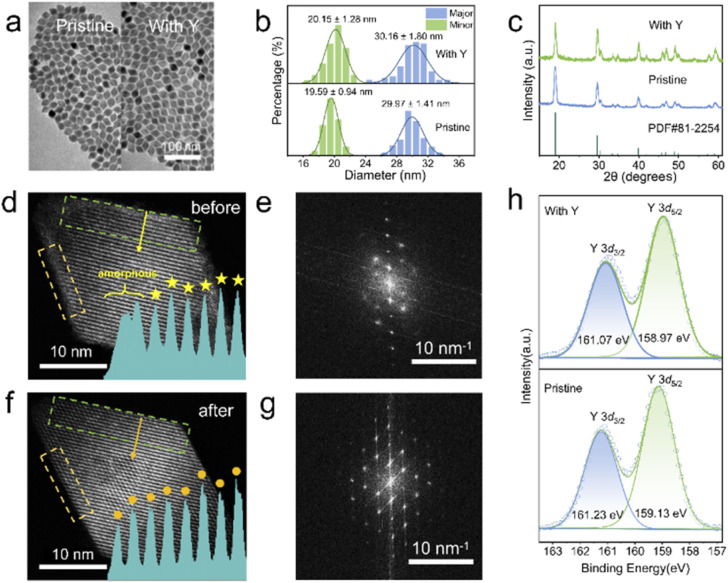
Revealing the evolution of lattice defects and structural reconstruction through AC-HAADF-STEM atomic-scale characterization. (a) TEM images of LiYF_4_:25%Yb^3+^,4%Tm^3+^ nanoparticles before and after Y^3+^ thermal annealing at 290 °C. (b) Histogram showing the size distribution of nanocrystals. (c) X-ray diffraction (XRD) patterns of core nanocrystals. (d and f) AC-HAADF-STEM images of the sample before (d) and after (f) treatment, with insets showing the intensity profile along the direction indicated by the arrows. (e and g) Fast Fourier Transform (FFT) patterns corresponding to the nanoparticles before (e) and after (g) treatment. (h) XPS spectra of LiYF_4_:Yb,Tm samples before and after treatment.

To investigate the surface lattice defects, we synthesized a series of LiYF_4_ nanoparticles *via* a coprecipitation method and performed wet-chemical annealing treatments with the assistance of F^−^, Li^+^, and Y^3+^ ions, respectively. The changes of morphology, size, and luminescence properties show significant differences when treated with different additives. After Li^+^ and F^−^-assisted treatment, the morphology of LiYF_4_ UCNPs changes from octahedral to spherical, with a slight reduction in size and a significant decrease in luminescence intensity (Fig. S2 and S3), indicating that the addition of Li^+^ and F^−^ ions cannot repair the surface lattice defects. Mechanistically, the introduction of exogenous Li^+^ and F^−^ ions promotes the preferential formation of a LiF phase from LiYF_4_ nanocrystals within a short period of time, thereby leading to a reduction in particle size.^35^ In contrast, Y^3+^-assisted treatment results in significantly enhanced emission intensity and enlarged lifetime with a uniform morphology and unchanged size (Fig. S4). For comparison, we also performed pure wet-chemical annealing without any additives. The results reveal that thermal annealing alone only leads to a slight enhancement in luminescence (Fig. S5). It is indicated that the introduction of Y^3+^ ions during annealing treatment can effectively eliminate surface lattice defects and improve optical performance. To further evaluate the elimination of surface vacancy defects through Y^3+^-assisted annealing at the atomic scale, we compared the basic structural variation of LiYF_4_:Yb,Tm UCNPs before and after treatment. The transmission electron microscopy (TEM) results indicated that the morphology and size of these nanoparticles showed no significant changes before and after treatment (Fig. 2a), and the size distribution remained uniform (Fig. 2b). The X-ray diffraction (XRD) patterns confirmed that the treatment would not alter the phase composition of these samples, but the sharpened diffraction peaks indicate that the annealing treatment can effectively improve the crystallinity (Fig. 2c).

Then, AC-HAADF-STEM was employed to explore the atomic-scale structural evolution before and after annealing (Fig. 2). The TEM image of the pristine nanoparticles (Fig. 2d) shows uniform lattice fringes throughout the interior region, which is free from grain boundaries or impurity phases. However, the relaxation-type intensity distribution observed at the periphery of the pristine nanoparticles (inset in Fig. 2d), quantified through characteristic peak-valley intensity extraction, directly correlates with surface lattice defects arising from incomplete crystallization during synthesis. And these defects and the outermost amorphous layer (yellow area in Fig. 2d) are mainly regarded as lanthanide vacancy induced defects.^34^ As can be seen from Fig. 2f, the intensity curve exhibits significantly enhanced periodic characteristics after Y^3+^-assisted annealing, which indicates the effectively elimination of surface lattice defects and reconstruction of lattice structure. Thus, the non-radiative transition pathways induced by surface quenching sites can be greatly suppressed. Besides, the defect distribution (marked in green) in Fig. S6a shows that the lattice defects are mainly concentrated in the surface edge region. As shown in Fig. 2d and S7, the pristine nanoparticles with different sizes all exhibit a surface defect layer of approximately 0.67 nm (about two atomic layers), which can be reconstructed after Y^3+^-assisted annealing (Fig. 2f and S6b).^36^ What's more, the corresponding FFT results (Fig. 2e and g) also demonstrate that the annealing treated nanoparticles exhibit sharper diffraction spots along the [0 1 0] zone axis, indicating improved crystallinity.

To verify the role of Y^3+^ in eliminating surface vacancy defects from an electronic structure perspective, we further performed X-ray photoelectron spectroscopy (XPS) before and after annealing. As shown in Fig. 2h, the Y^3+^ binding energy shows a negative shift (0.16 eV) after annealing. Essentially, the binding energy reflects the binding capacity of the atomic nucleus for outer-shell electrons, and its value exhibits a negative correlation with the electron density surrounding the ions.^37^ The decrease in the binding energy of Y^3+^ directly demonstrates a remarkable enhancement in the electron density around Y^3+^ ions, which originates from the effective elimination of cation vacancy defects on the surface of nanocrystals. The presence of vacancy defects gives rise to the inhomogeneous distribution of local electron clouds and low electron density. Nevertheless, the exogenously introduced Y^3+^ can occupy the lattice vacancies, thereby reducing the non-directional dispersion and outward distribution of electrons. This further elevates the local electron density around the ions, weakens the binding energy of outer-shell electrons, and ultimately manifests as a negative shift of the binding energy. Notably, compared with annealing treatment with and without additional Y^3+^ ions, the Y^3+^-assisted annealing nanoparticles exhibit a large reduction in binding energy (Fig. S8), revealing that the introduction of Y^3+^ can further optimize the local electronic structure and effectively promote the elimination of surface vacancy defects. Moreover, to exclude the interference of surface attached Y^3+^ ions, we further introduced Lu^3+^ ions during annealing to specifically monitor the valence state of Y^3+^ ions (Fig. S8). As for the Lu^3+^-assisted annealed nanoparticles, the binding energies of the Y 3d_5/2_ and Y 3d_3/2_ peaks shifted significantly toward the lower energy end by 0.46 eV compared to the pristine sample. This shift indicates an increase in the electron density of the outer shell of Y^3+^ within the nanoparticles, which is consistent with the charge compensation effect caused by the reduction of cation vacancies. Thus, the introduction of Y^3+^ can effectively fill the cation vacancies and reconstruct the lattice structure, thereby enhancing the upconversion emission.

**Fig. 3:**
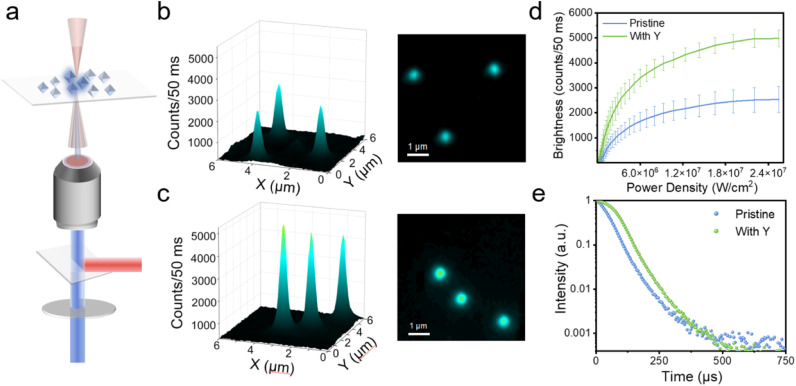
The *in situ* optical properties of a LiYF_4_:25%Yb^3+^,4%Tm^3+^ single nanoparticle before and after annealing treatment. A 4 mol% Tm^3+^ doping concentration was selected for single-particle high-power excitation to ensure a measurable upconversion emission signal.^38^ (a) Schematic diagram of a home-made laser confocal microscopy system. (b and c) Point scanning confocal microscopy images of nanoparticles before and after annealing treatment at a power density of 2.5 × 10^7^ W cm^−2^. (d) Power-dependent emission intensity curves. (e) Decay curves at 793 nm of nanoparticles before and after annealing treatment.

Normally, the optical performances of UCNPs are measured using solution, powder, or film systems. However, the precise quantity and concentration of UCNPs are difficult to keep exactly the same. What's more, the measured data are mainly macroscopic statistical averages, which mostly ignore the individual differences.^5,39^ To address these issues, we monitored the optical performance variation before and after annealing at the single-nanoparticle level. First of all, the fully diluted UCNPs were drop-cast onto glass coverslips to ensure that the distance between two nanoparticles is larger than the imaging resolution of the optical system.^40^ Then, the emission intensity and decay curve of each nanoparticle were measured using a home-made laser confocal microscopy system (Fig. 3a and S9).^10^Fig. 3b and c show the point scanning confocal microscopy images of UCNPs before and after annealing. The average emission intensity of individual nanoparticles before and after annealing treatment shows a significant enhancement, which increased from 2686 counts/50 ms to 4896 counts/50 ms (under an excitation power density of 2.5 × 10^7^ W cm^−2^), an approximately twofold enhancement (Fig. S10). The single-nanoparticle-based measurement effectively eliminated the potential errors introduced by macroscopic statistical averaging (such as concentration and uniformity) in solution-based measurements. To quantitatively evaluate the emission enhancement, we measured the excitation power-dependent emission intensity curves for 10 individual nanoparticles before and after annealing over an excitation power density range from ∼20 to 2.5 × 10^7^ W cm^−2^. As shown in Fig. 3d, the annealed nanoparticles exhibited enhanced emission over the entire power range, and the enhancement factor decreased gradually with increasing excitation power. At a power density of approximately 2.5 × 10^7^ W cm^−2^, the average emission intensity was enhanced to approximately twice that of the pristine sample. Furthermore, the lifetime of single nanoparticles also increased from 46 µs to 57 µs (Fig. 3e), demonstrating that the annealing treatment can effectively suppress defect-induced nonradiative transition. The optical performance at the single nanoparticle level is mutually corroborated with structural analyses at the atomic level, which further confirmed the important role of Y^3+^-assisted annealing treatment in reconstructing the surface lattice and improving optical performance. However, it should be noted that the single-nanoparticle measurements are mainly conducted under high-power excitation conditions, where the emitting centers are prone to excitation saturation, and the influence of surface quenching is relatively relieved.^38,41,42^ As a result, the direct contribution of surface defect elimination to the observed emission enhancement appears relatively modest under such conditions. Then, further investigation of the effect of annealing on optical behavior under low-power excitation was employed to reveal the role of surface defects more comprehensively, providing valuable complementary insights.

**Fig. 4:**
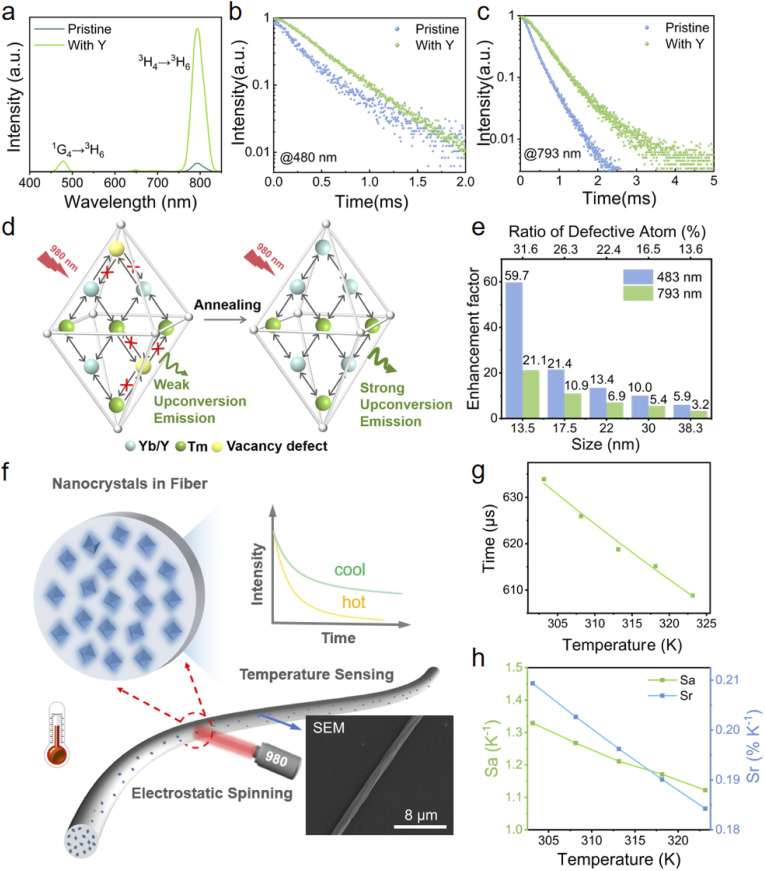
Annealing treatment induced optical performance improvement and temperature sensing application of treated UCNPs. LiYF_4_:25%Yb^3+^,0.5%Tm^3+^ nanoparticles were measured under low-power excitation to minimize Tm^3+^ concentration quenching.^43,44^ (a) Upconversion emission spectra of pristine and Y^3+^ ion assisted annealed LiYF_4_:25%Yb^3+^,0.5%Tm^3+^ nanoparticles under 980 nm excitation (1 W cm^−2^). Dynamic curves of Tm^3+^ at (b) 480 nm (^1^G_4_ → ^3^H_6_) and (c) at 793 nm (^3^H_4_ → ^3^H_6_). (d) Mechanism of luminescence enhancement by vacancy elimination. (e) Size dependent upconversion emission enhancement factors of LiYF_4_:25%Yb^3+^,0.5%Tm^3+^ nanoparticles before and after annealing treatment. (f) Schematic diagram of a lifetime mode temperature sensor. (g) Temperature-dependent trend of the fitted decay lifetimes under 980 nm excitation (monitored at 793 nm). (h) Relative sensitivity (*S*_r_) and absolute sensitivity (*S*_a_) *versus* the absolute temperature.

The optical performance of UCNPs with various doping and sizes before and after surface lattice reconstruction was further systematically investigated. It's known that the nanoparticles with a small size have a relatively high surface atomic occupancy rate. We herein selected a typical small upconversion nanoparticle for further exploration. The size (approximately 14 nm) and morphology of LiYF_4_:Yb,Tm nanoparticles (Fig. S11) remain unchanged after Y^3+^-assisted annealing treatment. Then, the upconversion emission spectrum and decay curve were measured. As shown in Fig. 4a, the emission intensity of the blue band (480 nm, ^1^G_4_ → ^3^H_6_) and near-infrared band (793 nm, ^3^H_4_ → ^3^H_6_) increased by 59.7 times and 21.1 times, respectively. The emission at 480 nm originates from the ^1^G_4_ → ^3^H_6_ transition *via* a three-photon upconversion process, while the emission at 793 nm corresponds to the two-photon ^3^H_4_ → ^3^H_6_ transition (Fig. S12). Transition pathways involving more photons are more strongly suppressed by surface lattice defects. Consequently, their luminescence performance is improved more significantly after annealing treatment. Further dynamic curves show that the lifetimes of ^1^G_4_ → ^3^H_6_ and ^3^H_4_ → ^3^H_6_ transitions extend from 298 µs and 313 µs to 427 µs and 582 µs, respectively (Fig. 4b and c). The enhanced emission intensity and enlarged lifetime both reveal that the Y^3+^-assisted annealing treatment can effectively eliminate surface lattice defects, thereby suppressing non-radiative transitions (Fig. 4d). In order to explore the influence of the surface lattice defect proportion on optical performance, we conducted Y^3+^-assisted annealing on LiYF_4_:Yb,Tm UCNPs with size ranging from 13.5 nm to 38.8 nm. The volume proportion of surface defects (two outermost atomic layers) increases from 13.6% to 31.6% as the particle size decreases from 38.8 nm to 13.5 nm (see the SI for a detailed calculation process). Importantly, the enhancement in emission intensity increases from 5.9-fold to 59.7-fold as the size decreases (Fig. 4e), which confirms that the reconstruction of the surface lattice is vital for improving the optical performance of small UCNPs.

Furthermore, we also explored the potential microscopic sensing applications by using the annealed UCNPs. These small but bright UCNPs were mixed uniformly with polystyrene to obtain the UCNP-doped electro-spun fibers through a high-voltage electrostatic field-driven microfluidic jetting technique (Fig. 4f, S13 and S14). Regardless of nanoparticle incorporation, the fiber diameter remains stable at 1.0 ± 0.2 µm (Fig. 4f and S15), demonstrating that the integration of UCNPs had no significant impact on the overall morphology of fibers. Then, the point scanning confocal microscopy image (Fig. S16) further confirmed the homogeneous optical emission, indicating the uniform distribution of UCNPs within the electro-spun fibers. Subsequently, the temperature dependent lifetime measurement was conducted on the electro-spun fibers (Fig. 4g and h). As the temperature increased from 303.15 K to 323.15 K, the near-infrared band emission lifetime corresponding to the ^3^H_4_ → ^3^H_6_ transition decreased from 634 µs to 609 µs (Fig. S17). Notably, due to the suppression of non-radiative relaxation caused by surface defects, the lifetime variation range is considerably larger than that of the untreated sample (Fig. S18). The maximum *S*_r_ is 0.21% K^−1^, and the *S*_a_ reaches 1.33 K^−1^ at 303.15 K. The correlation between the lifetime and temperature suggests the potential for temperature-sensing applications, particularly in microscale flexible biological systems, where the enhanced spatial resolution can significantly improve temperature measurement accuracy.

## Conclusions

In conclusion, a lanthanide ion-assisted annealing strategy was proposed to eliminate the surface lattice defects, and the upconversion optical performance improvement was demonstrated at the single-nanoparticle level. It was found that the Y^3+^ ions can preferentially fill the cationic vacancies, effectively removing surface lattice defects and reconstructing the lattice structure of LiYF_4_:Yb,Tm nanoparticles. Then, the detrimental energy transfer from the excited state to surface quenching sites would be blocked, thereby substantially enhancing the upconversion emission intensity. The size dependent improvement further demonstrated the possibility of obtaining small but bright UCNPs through a lanthanide ion assisted annealing strategy. As the nanocrystal size reduces from 38.8 nm to 13.5 nm, the proportion of surface defects (about two outermost atomic layers) increases from 13.6% to 31.6%. After Y^3+^ ion assisted annealing treatment, the corresponding emission intensity enhancement factor increases from 6 to 60-fold (^1^G_4_ → ^3^H_6_), indicating that the removal of surface lattice defects is particularly critical for improving the optical performance of small UCNPs. The surface lattice reconstruction strategy offers a new viable *in situ* approach to improve the optical performance of UCNPs without increasing the particle size.

## Experimental

Detailed experimental procedures are reported in the SI.

## Author contributions

Yunfei Shang conceptualized the project, conducted formal data analysis, provided essential resources, acquired financial funding, supervised the research work, and revised the manuscript. Fenglin Wang performed the investigation, curated the experimental data, visualized the results, and wrote the original draft of the manuscript. Xiaoyong Huang carried out the investigation and validated the experimental results. Jun Zeng contributed to result visualization. Xiangyu Pan participated in the experimental investigation. Fei Han, Yongtao Liu and Shuwei Hao supervised the research project; Shuwei Hao also provided essential resources and acquired funding support. Chunhui Yang supplied the necessary experimental resources. Jiajia Zhou conceptualized the project and reviewed and edited the manuscript.

## Conflicts of interest

The authors declare no conflict of interest.

## Supplementary Material

SC-OLF-D6SC03950B-s001

## Data Availability

The data that support the plots within this paper and other findings of this study are available from the corresponding author upon request. Source data are provided in this paper. Supplementary Information (SI): TEM images; AC-HAADF-STEM images; particle size distribution histograms; SEM images; XPS spectra; luminescence spectra; fluorescence decay curves, *etc.* See DOI: https://doi.org/10.1039/d6sc03950b.
