# Supplementary material for: Identification of surface defects and in situ lattice reconstruction of upconversion nanoparticles
Source: Chem Sci. 2026 Jul 27. Online ahead of print. doi: 10.1039/d6sc03950b (PMC13403942; doi:10.1039/d6sc03950b)
Supplement: SC-OLF-D6SC03950B-s001 [file SC-OLF-D6SC03950B-s001.pdf]

Supplementary Information (SI) for Chemical Science.
This journal is © The Royal Society of Chemistry 2026

Supporting Information

Identification of Surface Defects and in-situ Lattice Reconstruction of Upconversion Nanoparticles

Fenglin Wang[a], Xiaoyong Huang[a], Yunfei Shang[a,b *], Jun Zeng[a], Yongtao Liu[c], Shuwei Hao[a], Chunhui Yang[a], Jiajia Zhou[d,*]

[a] MIIT Key Laboratory of Critical Materials Technology for New Energy Conversion and Storage, School of Chemistry and Chemical Engineering, Harbin Institute of Technology, Harbin 150001, China

[b] Zhengzhou Research Institute of Harbin Institute of Technology, Zhengzhou, 450001, China

[c] School of Electronic and Optical Engineering, Nanjing University of Science and Technology, Nanjing 210094, Jiangsu, China

[d] Institute for Biomedical Materials & Devices (IBMD), Faculty of Science, University of Technology Sydney, Sydney, NSW 2007, Australia

*Corresponding Author

E-mail: shangyunfei@hit.edu.cn (Yunfei Shang), Jiajia.Zhou@uts.edu.au (Jiajia Zhou)

ORCID: 0000-0003-1752-4815 (Yunfei Shang), 0000-0002-0605-5745 (Jiajia Zhou)

Materials: Oleic acid (OA, AR), 1-octadecene (ODE, >90%), methanol (>99.8%), cyclohexane (>99.7%), ethanol (water≤0.3%), N, N-dimethylformamide (DMF, >99.8%), yttrium chloride hexahydrate ($YCl_3 \cdot 6H_2O$; 99.9%), ytterbium chloride hexahydrate ($YbCl_3 \cdot 6H_2O$; 99.9%), thulium chloride hexahydrate ($TmCl_3 \cdot 6H_2O$; 99.9%), lithium hydroxide ($LiOH \cdot H_2O$; >99%), ammonium fluoride (NH_4F; >98%), polystyrene (PS, >98%) and nitrosonium tetrafluoroborate ($NOBF_4$; 95%) were all purchased from Aladdin Reagents Co. Ltd and used as received without further purification.

Synthesis of $LiYF_4$:25%Yb^{3+},0.5%Tm^{3+} UCNPs: The rare-earth chlorides ($YCl_3 \cdot 6H_2O$, 0.745 mmol; $YbCl_3 \cdot 6H_2O$, 0.25 mmol; $TmCl_3 \cdot 6H_2O$, 0.005 mmol) were added to a 250 mL three-necked flask containing OA (10 mL) and ODE (10 mL) at room temperature with stirring. The mixture was heated to 160°C under argon for 30 min, yielding a transparent solution and removing water. After cooling to room temperature, a methanol solution (10 mL) containing $LiOH \cdot H_2O$ (2.5 mmol) and NH_4F (4 mmol) was added dropwise, and the mixture was stirred for 30 min. The temperature was subsequently raised to 80°C for 30 min to evaporate methanol and then to 290°C for 1.5 h under argon. The cooled $LiYF_4$:25%Yb^{3+},0.5%Tm^{3+} nanoparticles was purified via three cycles of dissolution/precipitation (cyclohexane/ethanol) and stored in 10 mL of cyclohexane. $LiYF_4$:25%Yb^{3+},4%Tm^{3+} nanoparticles were prepared analogously by adjusting the precursor ratios.

Synthesis of Ultra Small $LiYF_4$:25%Yb^{3+}, 0.5%Tm^{3+} UCNPs: By slightly modifying the method reported by Hyeon's group[1], 1 mmol of rare-earth oleate ($RE(oleate)_3$, where RE is Y, Yb, and Tm with molar ratios of 74.5%, 25%, and 0.5%, respectively) was synthesized. Subsequently, $LiYF_4$:25%Yb^{3+},0.5%Tm^{3+} UCNPs were synthesized by a coprecipitation method[2, 3]. Specifically, 1 mmol of $RE(oleate)_3$ was mixed with OA (10 mL) and ODE (10 mL) in a three-necked flask. The mixture was heated to 150°C and maintained for 30 min before being cooled to 50°C. Then, a MeOH solution (10 mL) containing $LiOH \cdot H_2O$ (2.5 mmol) and NH_4F (4 mmol) was injected into the flask. After removing the MeOH solvent by evaporation, the reaction mixture was heated to 290°C and maintained for 1.5 h. Finally, the cooled product was purified via three cycles of dissolution/precipitation (cyclohexane/ethanol) and stored in 10 mL of cyclohexane.

Wet chemical annealing of $LiYF_4:Yb^{3+}$, Tm^{3+} UCNPs: Add $YCl_3{\cdot}6H_2O$ (0.3, 0.45, and 0.6 mmol) to three separate three-neck flasks, each containing OA (3 mL) and ODE (3 mL). The mixture was stirred and heated to 160°C under an argon atmosphere and maintained at this temperature for 30 min, resulting in a precursor solution. Subsequently, 6 mL of this precursor solution was mixed with 3 mL of a nanoparticle cyclohexane dispersion. The combined mixture was held at 160°C for 30 min to evaporate cyclohexane and remove residual moisture. The temperature was then raised to 290°C and maintained for 30 min. After cooling to room temperature, the product was isolated by centrifugation (8000 r/min, 5 min) following the addition of ethanol. This purification cycle was repeated 2-3 times. The purified product was dispersed in cyclohexane for storage.

The ligand removal method for nanoparticles: To remove the OA ligands, $LiYF_4$:25%Yb^{3+},0.5%Tm^{3+} UCNPs (in 1 mL of hexane) were subjected to a ligand exchange process. Specifically, a solution of $NOBF_4$ (0.015 g) in DMF (1 mL) was prepared and sonicated for 2 min to ensure complete dissolution. This solution was then mixed with the nanoparticle dispersion and shaken for 10 min to remove the surface OA ligands. Subsequently, hexane (5 mL) and toluene (5 mL) were added, and the mixture was allowed to stand for 10 min. The ligand-free nanoparticles were collected by centrifugation at 8000 rpm for 5 min and finally dispersed in DMF.

Preparation of electro-spun fibers: PS was dissolved in DMF to prepare a precursor solution for electrospinning with a concentration of 40 wt%. The solution was stirred at 500 rpm for 2 h at room temperature until complete dissolution. electro-spun fibers was then performed using a portable in-situ setup with an applied voltage of 15 kV and a controlled tip-to-collector distance. The resulting fibers were collected on a flat collector.

Sample preparation for single particle testing: First, dilute the nanoparticle dispersion to a concentration of 200 $ng{\cdot}mL^{-1}$. Then, drop 10 μL of the diluted solution onto a clean and dry glass slide that has been washed with ethanol. After the diluted solution has dried naturally, affix it to a standard microscope slide with adhesive, leaving a small window for observation[4].

Single particle imaging: Single particle optical characterization is performed on an

optical microscope system equipped with a UPLSAPO 100 X NA 1.40 oil immersion objective and a 976 nm fiber laser (BL 976-PAG 900, Thorlabs). The excitation light is reflected by a dichroic mirror (DM) integrated into the microscope and irradiates the nanoparticles through the objective lens. The emission signal from the nanoparticles is collected by the same objective lens, filtered by a bandpass filter, and then detected by an avalanche photodiode mounted on the microscope.

Correlative SEM and wide-field imaging: For the scanning electron microscope analysis of electro-spun fibers, a thin layer of gold is applied to the same samples to enhance conductivity, and field emission scanning electron microscopy (Zeiss Supra™ 55) was used for detection. The pre-marked patterns serve as navigation guides to locate the patterns that have previously undergone optical characterization. The morphology of the electro-spun fibers and the dispersion of the nanoparticles can be verified when the electron microscope patterns of these particles match the fluorescence images.

Characterization: The microstructure of the electro-spun fibers was characterized by SEM (Supra™ 55, Zeiss, Germany) at an acceleration voltage of 15 kV. The morphology and size of the nanoparticles were examined by TEM (Tecnai F20 G2, FEI, USA) operated at 100 kV. Point defects in the nanoparticles were analyzed using HAADF-STEM (JEM-ARM 300F, JEOL, Japan) at 200 kV. The crystal phase was determined by XRD (D8 ADVANCE, Bruker, Germany) with Cu K_{α} radiation ($K_{\alpha 1}$ = 1.5406 Å, $K_{\alpha 2}$ = 1.54439 Å, $K_{\alpha 1}/K_{\alpha 2}$ = 2) at 30 kV and 10 mA, using a scanning range of 10°–90° (2θ) with a step size of 0.02° and a dwell time of 0.15 s per step. The elemental valence states were investigated by XPS (AXIS ULTRA DLD, Shimadzu, Japan) with a monochromatic Al Kα X-ray source. Up-conversion luminescence spectra and lifetimes were recorded using a Fluorolog-3.11 fluorescence spectrometer (Jobin Yvon). Near-infrared (NIR) excitation light was vertically incident on a cuvette sample, and the up-conversion luminescence signal was collected at a direction perpendicular to it. All acquired luminescence spectra were corrected for instrumental response using calibration curves provided by the Fluorolog system.

Temperature sensitivity calculation: In this study, the up-conversion luminescence lifetime shortens with increasing temperature, primarily due to the sharp increase in the non-radiative relaxation rate with temperature. Based on the non-radiative relaxation theory model, the expression relating the fitted lifetime to temperature is as follows[5]:

$$\tau(T) = \frac{\tau_0}{Cexp\left(-\Delta E/_{k_BT}\right) + 1}\#(S - 1)$$

This equation includes the decay lifetime at real-time temperature (τ), the lifetime at the initial temperature (τ_0), the Boltzmann constant (k_B), and a constant (C).

The corresponding relative sensitivity (S_r) and absolute sensitivity (S_a) can be calculated using the following formulas[6, 7]:

$$S_a = \left|\frac{\partial \tau}{\partial T}\right|\#(S - 2)$$

$$S_r = \left|\frac{1}{\tau}\frac{\partial \tau}{\partial T}\right| \times 100\%\#(S - 3)$$

Calculation of the proportion of surface lattice defects in $LiYF_4$ nanoparticles: According to the results of HAADF-STEM testing, the thickness of the surface lattice defects is approximately 0.67 nm. Based on the octahedral characteristics of the material, the specific formula for calculating the proportion of defects is as follows[2, 8]:

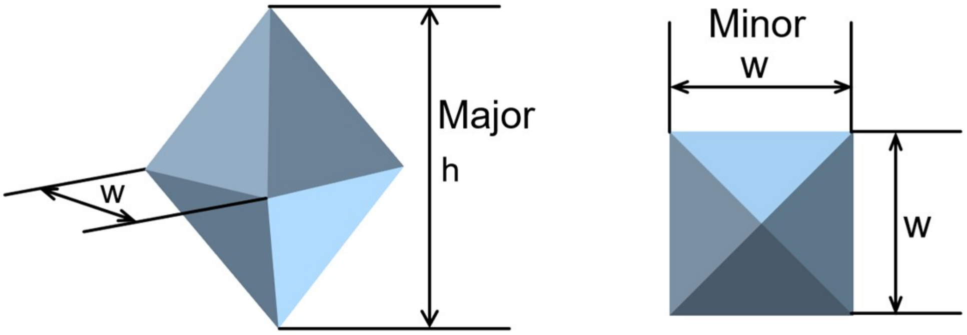

Figure S1. Schematic illustration of the octahedral structure of a $LiYF_4$ nanoparticle

$$\varphi = \frac{V_d}{V_w} \times 100\%\#(S - 4)$$

$$V_w = \frac{w^2h}{3}\#(S - 5)$$

$$V_d = \frac{w^2h - (w - 2d)^2(h - 2d)}{3}\#(S - 6)$$

where φ denotes the defect proportion (%); V_d and V_w denote the volumes (nm^3) of the surface lattice defect zone and the whole nanoparticle, respectively; w and h represent the characteristic dimensions (nm) defined in the octahedron of Fig. S1; and d is the thickness of the surface lattice defects, taken as 0.67 nm.

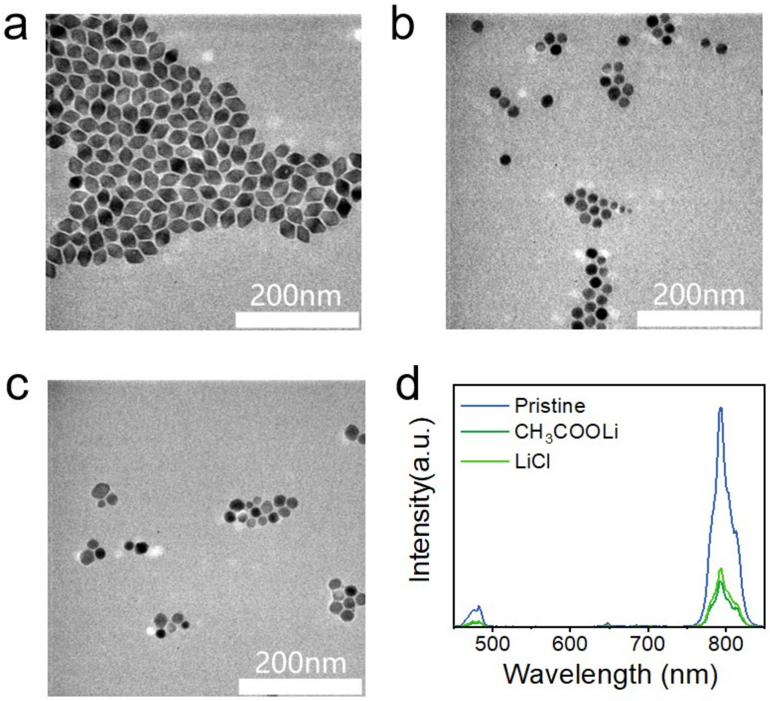

Figure S2. Effect of Li^{+} addition on the annealing treatment of nanoparticles. TEM images of $LiYF_4$:25%Yb^{3+},0.5%Tm^{3+} nanoparticles (a) before and (b) after wet-chemical annealing show a reduction in both particle size and morphological definition. (c) The corresponding luminescence spectra indicate a significant decrease in emission intensity after treatment.

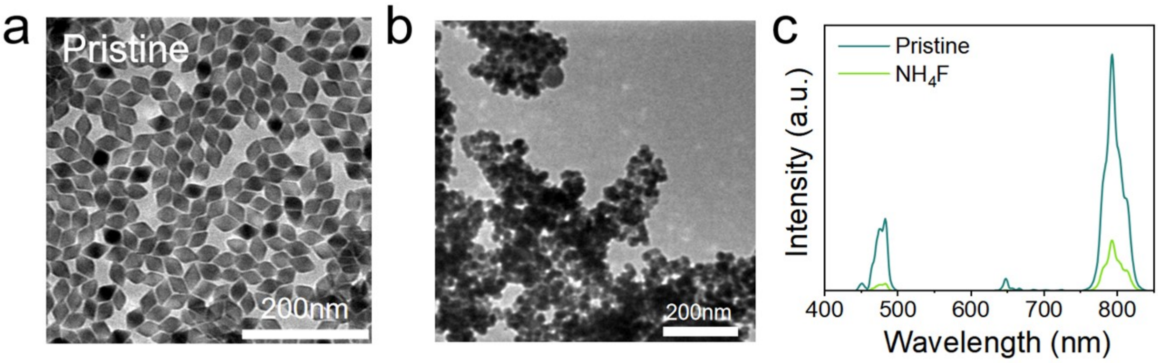

Figure S3. Effect of F^{-} additive on the annealing treatment of nanoparticles. TEM images of $LiYF_4$:25%Yb^{3+},0.5%Tm^{3+} nanoparticles (a) before and (b) after wet-chemical annealing show morphological degradation and severe aggregation. (c) The corresponding luminescence spectra indicate a significant reduction in emission intensity after treatment.

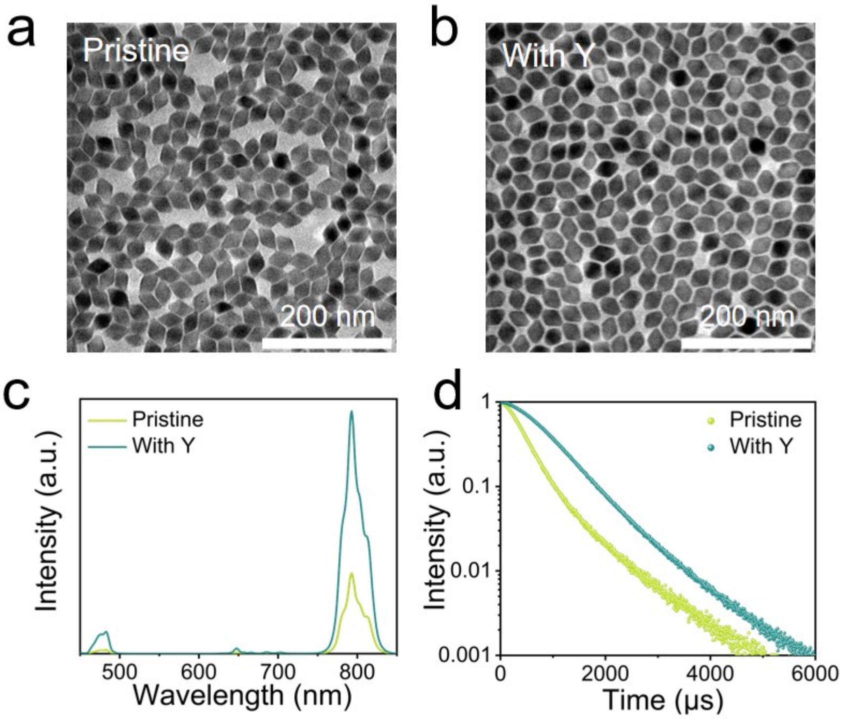

Figure S4. **Effect of annealing with Y^{3+} addition on the morphology and luminescence properties of $LiYF_4$:25%Yb^{3+}, 0.5%Tm^{3+} nanoparticles.** TEM images (a, b) indicate that the particle morphology and size remain essentially unchanged before and after annealing. The corresponding emission spectra (c) and fluorescence decay curves (d) show that after annealing, the luminescence intensity is significantly enhanced and the fluorescence lifetime is notably prolonged.

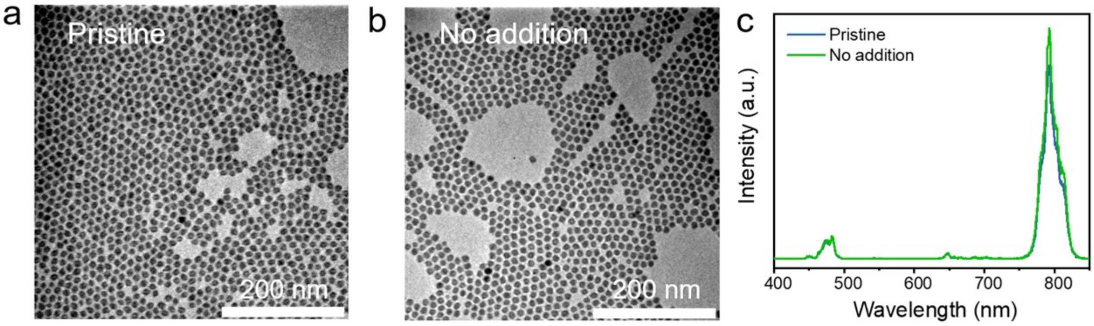

Figure S5. **Effect of no additive on the annealing treatment of nanoparticles.** TEM images of $LiYF_4$:25%Yb^{3+},0.5%Tm^{3+} nanoparticles (a) before and (b) after wet-chemical annealing. (c) The corresponding luminescence spectra indicate a slight increase in emission intensity after treatment.

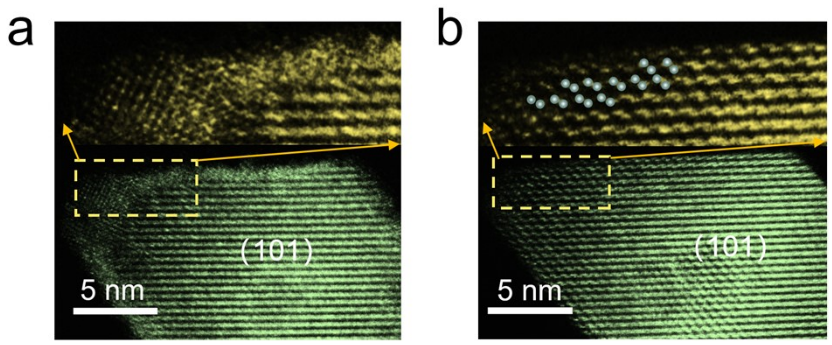

Figure S6. AC-HAADF-STEM images of the sample before (a) and after (b) treatment, with insets showing the intensity profile along the direction indicated by the arrows.

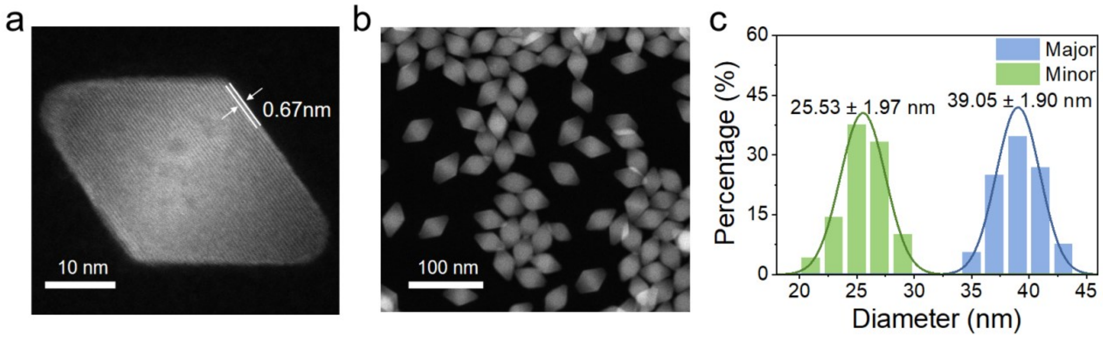

Figure S7. Microstructure and size distribution of LiYF$_4$: 25%Yb^{3+}, 4%Tm^{3+} UCNPs. (a) High-resolution AC-HAADF-STEM image, where a defect layer of ~0.67 nm is observed. (b) Low-resolution AC-HAADF-STEM image of the same region shown in (a). (c) Particle size distribution histogram.

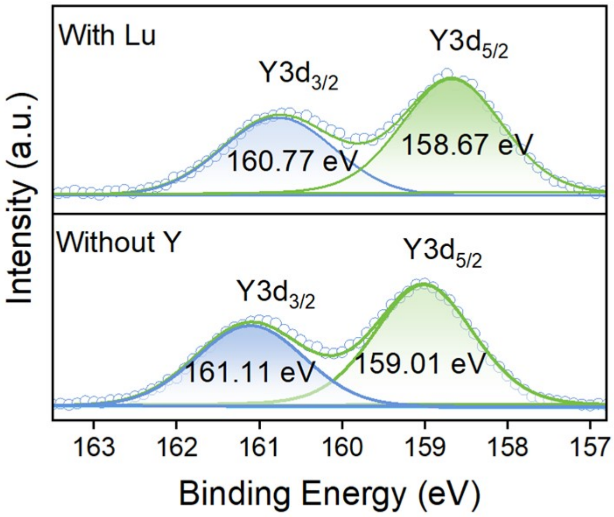

Figure S8. XPS spectrum of LiYF$_4$:25%Yb,0.5%Tm samples under different treatment conditions.

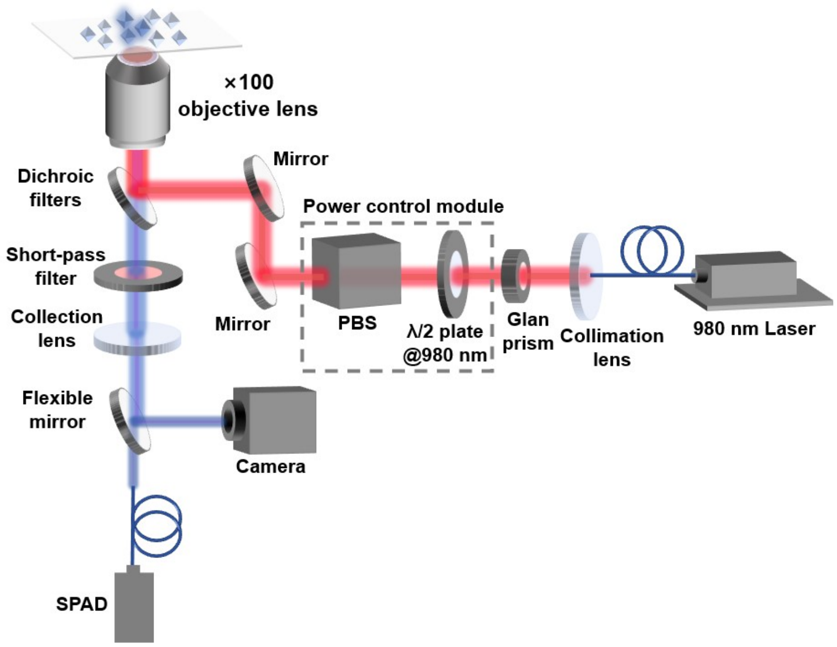

Figure S9. Schematic diagram of the single-particle detection system

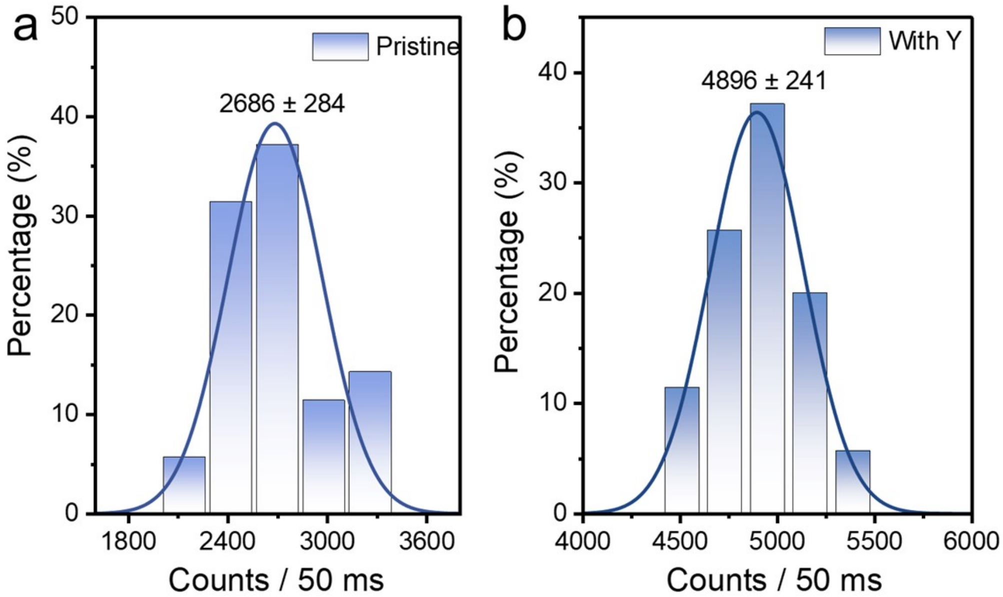

Figure S10. Brightness distribution before (a) and after (b) treatment under an excitation power density of 2.5×10^7 W/cm²

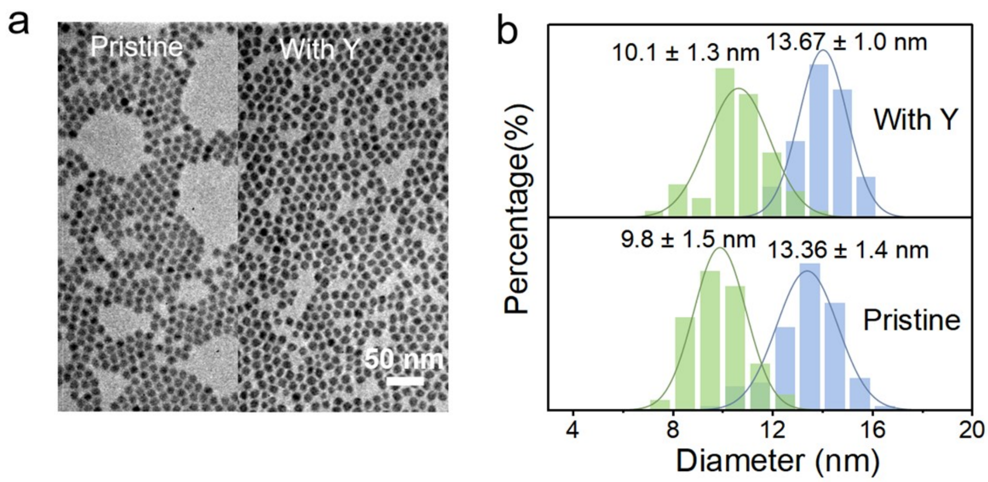

Figure S11. (a) Transmission electron microscopy (TEM) images of $LiYF_4$:25%Yb,4%Tm nanoparticles before and after Y^{3+} thermal annealing at 290℃. (b) Histogram showing the size distribution of nanocrystals.

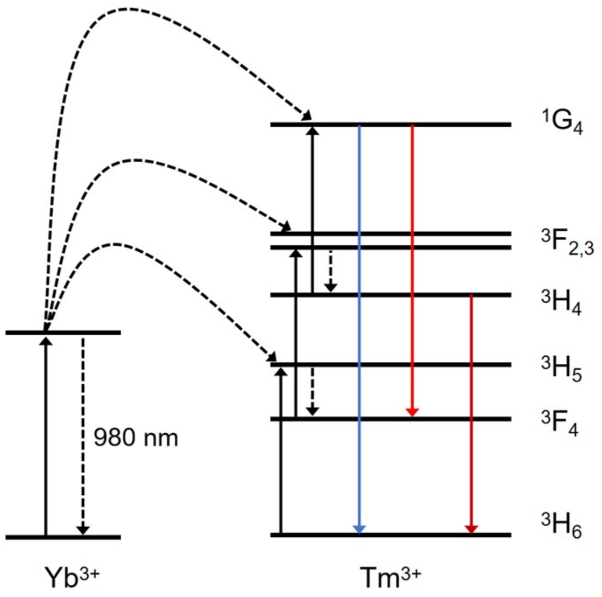

Figure S12. Schematic energy level diagram showing luminescence from $LiYF_4$:Yb/ Tm UCNPs.

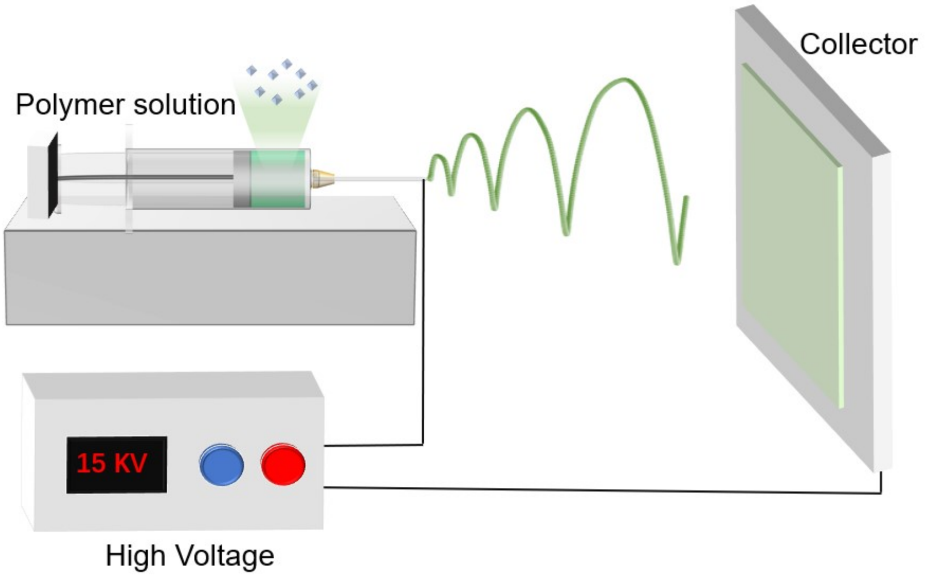

Figure S13. Schematic diagram of the electro-spun fibers apparatus.

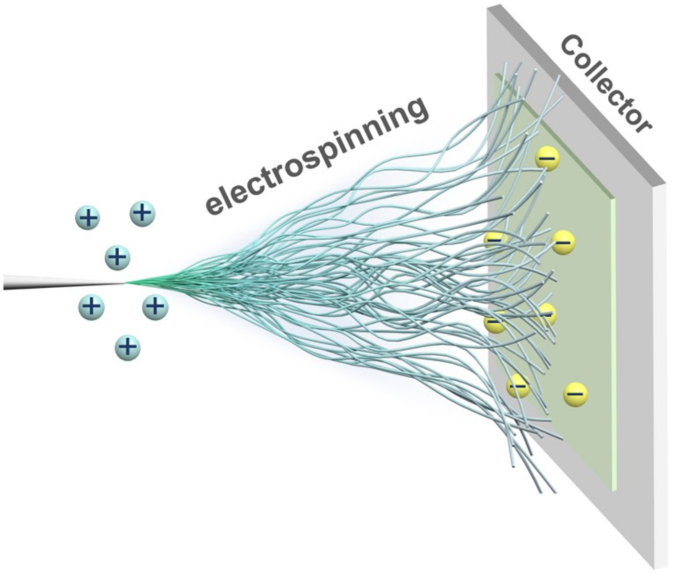

Figure S14. Schematic diagram of the preparation process for electro-spun fibers.

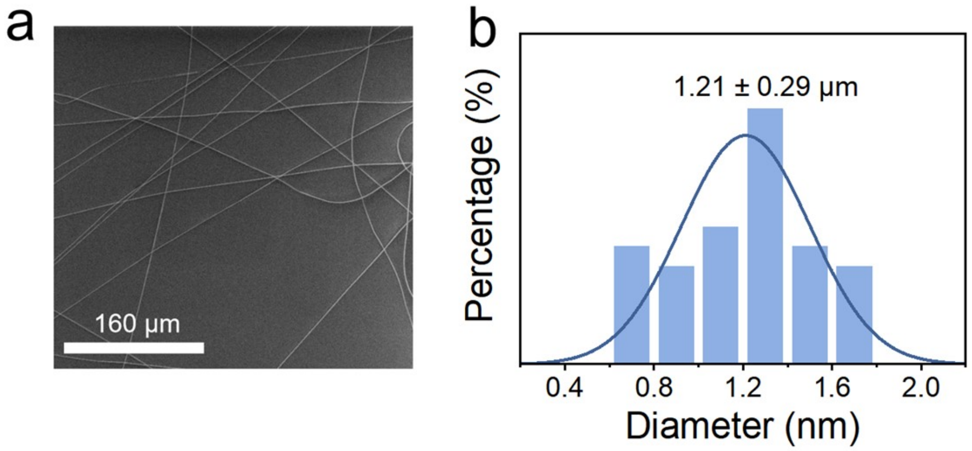

Figure S15. Microstructure and size distribution of electro-spun fibers. (a) SEM image. (b) Diameter distribution.

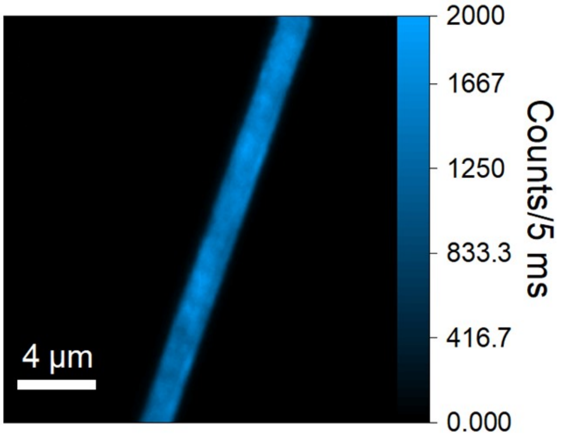

Figure S16. Optical micrograph of electro-spun fibers within a 20 μm×20 μm field of view.

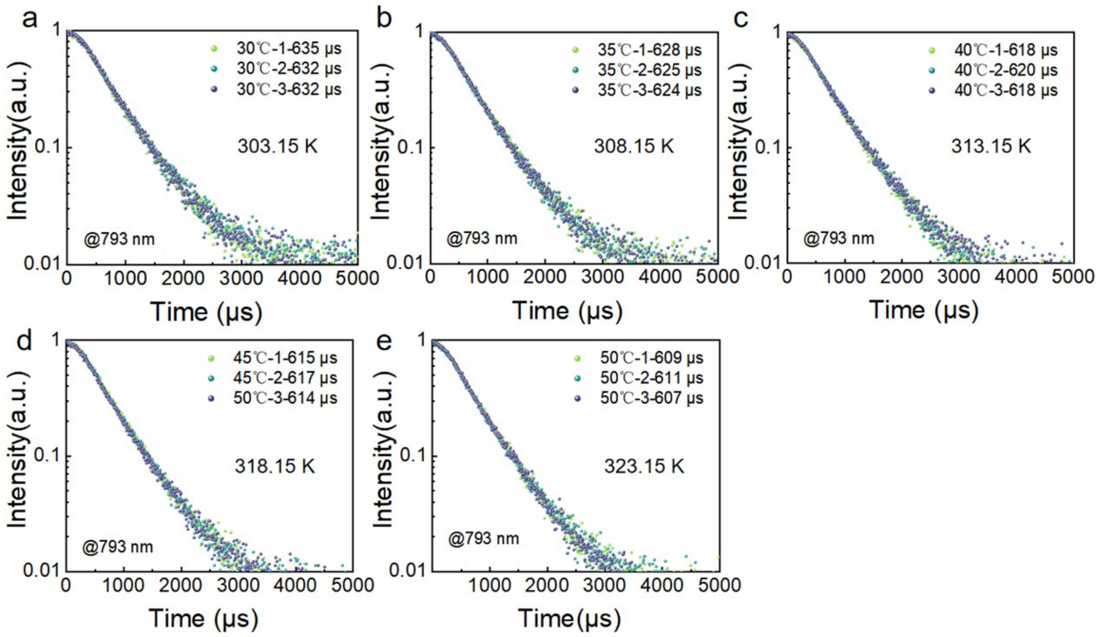

Figure S17. Temperature-dependent fluorescence lifetime of nanocrystals embedded in electrospun fibers at (a) 303.15 K, (b) 308.15 K, (c) 313.15 K, (d) 318.15 K, and (e) 323.15 K.

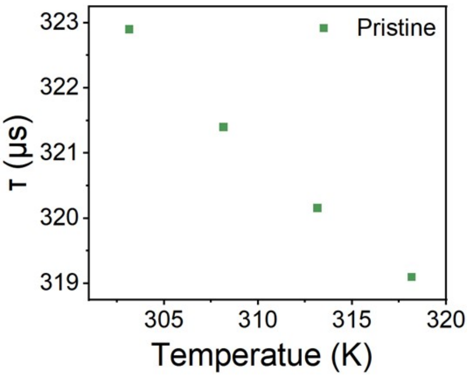

Figure S18. Temperature-dependent fluorescence lifetime curves of pristine nanocrystals embe dded in electrospun fibers at (a) 303.15 K, (b) 308.15 K, (c) 313.15 K and (d) 318.15 K.

Reference

1. J. Park, K. An, Y. Hwang, J. G. Park, H. J. Noh, J. Y. Kim, J. H. Park, N. M. Hwang and T. Hyeon, *Nat. Mater.*, 2004, **3**, 891-895.
2. H. Na, J. S. Jeong, H. J. Chang, H. Y. Kim, K. Woo, K. Lim, K. A. Mkhoyan and H. S. Jang, *Nanoscale*, 2014, **6**, 7461-7468.
3. J. Shin, J. H. Kyhm, A. R. Hong, J. D. Song, K. Lee, H. Ko and H. S. Jang, *Chem. Mater.*, 2018, **30**, 8457-8464.
4. Q. Liu, Y. Zhang, C. S. Peng, T. Yang, L. M. Joubert and S. Chu, *Nat. Photonics*, 2018, **12**, 548-553.
5. Y. Jiang, Y. Tong, S. Chen, W. Zhang, F. Hu, R. Wei and H. Guo, *Chem. Eng. J.*, 2021, **413**, 127470.
6. M. Zhang, B. Wang, Y. Cai, D. Jin and J. Zhou, *Nano Lett.*, 2024, **24**, 4877-4884.
7. L. Ma, F. Lu, Q. Yu, P. Dai, F. Hu, H. Guo and R. Wei, *Ceram. Int.*, 2023, **49**, 16681-16689.
8. N. Panov, D. Lu, E. Ortiz Rivero, E. Martinazzo Rodrigues, P. Haro González, D. Jaque and E. Hemmer, *Adv. Opt. Mater.*, 2021, **9**, 2100101.
